# Modeling Chronic Traumatic Encephalopathy: The Way Forward for Future Discovery

**DOI:** 10.3389/fneur.2015.00223

**Published:** 2015-10-26

**Authors:** Ryan C. Turner, Brandon P. Lucke-Wold, Aric F. Logsdon, Matthew J. Robson, John M. Lee, Julian E. Bailes, Matthew L. Dashnaw, Jason D. Huber, Anthony L. Petraglia, Charles L. Rosen

**Affiliations:** ^1^Department of Neurosurgery, West Virginia University School of Medicine, Morgantown, WV, USA; ^2^Center for Neuroscience, West Virginia University School of Medicine, Morgantown, WV, USA; ^3^Department of Basic Pharmaceutical Sciences, West Virginia University School of Pharmacy, Morgantown, WV, USA; ^4^Department of Pharmacology, Vanderbilt University School of Medicine, Nashville, TN, USA; ^5^Department of Pathology and Laboratory Medicine, NorthShore University Health System, University of Chicago Pritzker School of Medicine, Evanston, IL, USA; ^6^Department of Neurosurgery, NorthShore University Health System, University of Chicago Pritzker School of Medicine, Evanston, IL, USA; ^7^Department of Neurosurgery, University of Rochester School of Medicine and Dentistry, Rochester, NY, USA; ^8^Division of Neurosurgery, Rochester Regional Health, Rochester, NY, USA

**Keywords:** chronic traumatic encephalopathy, preclinical models, neurodegeneration, hyperphosphorylated tau, neurotrauma

## Abstract

Despite the extensive media coverage associated with the diagnosis of chronic traumatic encephalopathy (CTE), our fundamental understanding of the disease pathophysiology remains in its infancy. Only recently have scientific laboratories and personnel begun to explore CTE pathophysiology through the use of preclinical models of neurotrauma. Some studies have shown the ability to recapitulate some aspects of CTE in rodent models, through the use of various neuropathological, biochemical, and/or behavioral assays. Many questions related to CTE development, however, remain unanswered. These include the role of impact severity, the time interval between impacts, the age at which impacts occur, and the total number of impacts sustained. Other important variables such as the location of impacts, character of impacts, and effect of environment/lifestyle and genetics also warrant further study. In this work, we attempt to address some of these questions by exploring work previously completed using single- and repetitive-injury paradigms. Despite some models producing some deficits similar to CTE symptoms, it is clear that further studies are required to understand the development of neuropathological and neurobehavioral features consistent with CTE-like features in rodents. Specifically, acute and chronic studies are needed that characterize the development of tau-based pathology.

## Introduction

Corsellis described the original case series of chronic traumatic encephalopathy (CTE) in boxers (1). The disease consisted of brain atrophy, dilated ventricles, a cavum septum pellucidum, and pallor of the substantia nigra. CTE was reintroduced into the medical lexicon by Dr. Bennet Omalu in 2005 (2). Omalu described a progressive tauopathy that was seen in the brains of deceased football players. In recent years, McKee, Goldstein, and Stern have defined clinical and pathological features of the disease. These include behavioral disturbances such as impulsivity, depression, and lack of oversight (3). Pathological criteria include neurofibrillary tangles (NFTs) in a perivascular distribution and within superficial cortical areas with occasional amyloid and TDP-43 protein aggregations (4). Stern recently expanded the CTE criteria further by describing young versus old onset based on symptom manifestation (5). Interestingly, blast traumatic brain injury has been linked to CTE following a single exposure, where athletes develop the disease following repetitive head injury (6).

Chronic traumatic encephalopathy has been defined as a slowly progressive disease that takes years to decades to develop, often providing a significant latent period between when the neurotrauma occurs and when symptoms develop. A few cases involve athletes/soldiers as early as late teen’s to early 20s (7, 8). The reason for the discrepancies in age of presentation observed is currently unknown but is likely due to the age at which impacts were sustained and the severity of the injury. Prior studies have shown that children with TBI have inadequate development of social cognition (9) and that adolescents can develop post-traumatic headaches. Very rarely, however, do either of these groups experience symptoms of CTE (10). Early behavioral symptoms of CTE usually do not appear until the mid-30s, and cognitive impairment does not begin until the early sixties (4). Recent evidence suggests that neurotrauma may be linked to other neurodegenerative diseases such as Alzheimer’s disease (AD) as well (11).

Chronic traumatic encephalopathy and AD, despite both being tauopathies, have generally been viewed as separate diseases. Each disease has distinct clinical presentations and unique clinical risk values. This view has gradually been changing with some reports showing that CTE can develop within the context of AD. The relationship between these two conditions, however, is poorly understood. Prior studies have indicated that TBI is a risk factor for development of cognitive impairment and AD (12), but whether the conditions are additive or synergistic remains unclear (13). Recent evidence suggests that neurotrauma may both increase the likelihood of disease development and accelerate the development of AD (14). Notably, the wealth of AD pathology observed in preclinical neurotrauma models supports the idea of disease acceleration (15). In addition to AD, some groups have presented a possible link between CTE and a variant of amyotrophic lateral sclerosis that has been termed “chronic traumatic encephalomyelopathy” (CTEM) (16). Therefore, it is clear that neurotrauma may have many lasting deleterious consequences, including the potential for increased risk and accelerated development of neurodegenerative diseases such as AD, CTE, and CTEM. While the data on these studies are preliminary in nature and not prospective, these findings demonstrate the need for further investigation with both preclinical models and clinical trials.

## The Quest for the Ideal CTE Model

The search for ideal preclinical models to study CTE remains an area of ongoing investigation with a relative paucity of prior studies. Of those published previously, the majority of studies fail to recapitulate the extensive neuropathological and neurobehavioral aspects of injury (17, 18). Post-mortem identification of NFTs are a key diagnostic marker used clinically (19). Therefore, the development of hyperphosphorylated tau in animals following injury must be an important component in establishing a CTE model. Tau hyperphosphorylation appears to correlate with the emergence of neuropathological and neuropsychiatric deficits representative of neurotrauma-related neurodegeneration (20). By modeling CTE in rodents, we can better understand disease development and discover potential therapeutic avenues. Similarly, little justification is given to injury severity, number of impacts, interval between impacts, and age at which the impacts occur. Few studies have also evaluated secondary mechanisms of injury. In the following sections, we discuss the advancements and shortcomings of prior research while highlighting areas in need of further investigation.

### Proper Controls to Use When Studying CTE

**Figure 1:**
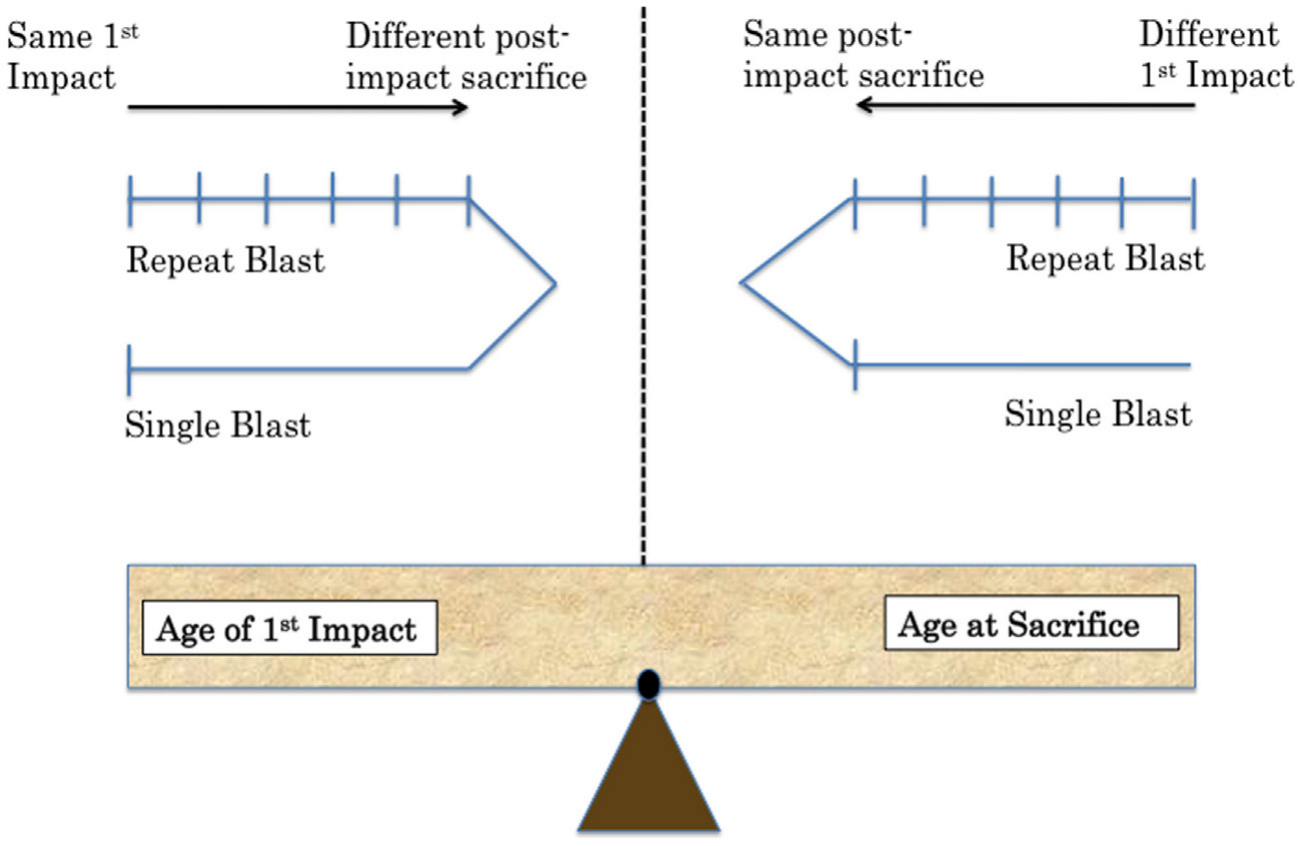
**Methodological challenges associated with repeat injury in comparison to single-injury paradigms include balancing equal age at time of exposure versus age at sacrifice**. Two possibilities are shown depending on the variable the experimenter wants to control in future work.

One of the challenges in creating a preclinical model for CTE is the establishment of proper controls comparing sham to TBI animals (13). Specifically, sham animals must undergo the exact same procedure each day of the injury paradigm. They must be anesthetized for the same duration as injured animals. The age of the animal must also be considered. Control animals should be the same age as experimental animals at time of sacrifice. This is an important consideration in designing extended studies with behavioral assays. Biochemical time courses also require control animals for each time point. Numerous questions must be addressed in planning experiments while considering the implications of each decision in terms of experimental question addressed and potential complications (Figure 1).

### Available Tools to Study CTE

One of the current weaknesses of clinical investigation of CTE is that the disease remains a neuropathological diagnosis. Some advancement has been made in ligand-based PET imaging. The imaging was used in a cohort of patients with an extensive history of neurotrauma, but the modality is not readily available (21). Consequently, clinical observations are largely retrospective in nature and it is nearly impossible to investigate disease onset and progression. These clinical shortcomings can be readily addressed through the use of adequately designed preclinical models.

The first step in creating a preclinical model of CTE is to choose a model that can be used to generate a combination of biochemical and behavioral changes post-injury consistent with the CTE-like phenotype. Specifically, tauopathy or a precursor of tauopathy (tau hyperphosphorylation), must be present. The changes in tau must be inducible in genetically unaltered animals and then must be verified with transgenic rodents. Behavioral changes must be induced by neurotrauma. These changes should persist or worsen at chronic time points post-injury. Assessment of behavior should include tests capable of evaluating behavioral symptoms reported in clinical CTE cases. In particular, tests for cognition, depressive-like behavior, and impulsive-like behavior should be used. Cognition can be measured using Morris water maze (MWM), whereas depressive-like behavior can be assessed with the forced swim test or tail suspension test. Impulsivity can be elucidated with the elevated-plus maze (EPM).

Once a model is established, biochemical, electrophysiological, and advanced imaging techniques can be employed to assess neural injury. Biochemical mechanisms of potential interest include those related to cell survival/death, regulation of tau phosphorylation/dephosphorylation (kinases/phosphatases), bioenergetics, and propagation of tau-based changes. Electrophysiological studies can be performed to identify effects of injury and CTE development both on individual synapses and larger tracts such as the Schaffer collaterals within the hippocampus. Imaging modalities, such as magnetic resonance imaging (MRI), functional MRI (fMRI), spectroscopy (MRS), and PET studies, have the unique advantage of being able to be performed longitudinally with multiple assessments of the same animal at different time points. Taken together, integration of biochemical, behavioral, electrophysiological, and imaging modalities may provide insight into the mechanisms and time course of CTE development. This approach will also allow for more definitive evidence to be gathered. This evidence will provide a stepping-stone in addressing key questions about the effect of inter-injury interval, injury severity, and number of cumulative injuries necessary for the development of CTE. The answers garnered from this potential work may then influence the design of clinical trials that dictate return-to-play decisions. Baseline monitoring may become required in sports arenas and battlefields with high incidences of neurotrauma. The monitoring may assist in detecting cumulative subthreshold injury levels and be used to decrease the overall level of concussions.

## Implications for Modeling CTE: Results from Prior CTE Modeling Studies

Perhaps one of the most promising studies, with regards to demonstrating CTE-like disease in a rodent model, was performed by Luo et al. In this study, the authors developed a closed-head model of neurotrauma utilizing an electromagnetic stereotaxic impact device. The authors showed that enhanced force of injury or using repeat injury increased GFAP-tagged luciferase. Intriguingly, when this same repeat-injury paradigm was applied to wild-type mice, spatial learning, and memory deficits were observed 2–6 months after injury and were accompanied by increased hyperphosphorylated tau and astrogliosis (22). The gliosis response in human CTE has not been well characterized, but the findings by Luo and colleagues show that GFAP was increased near areas of tauopathy. Similar findings have been seen by Petraglia and colleagues who performed a rigorous investigation of the behavioral effects of both single and repetitive closed-head injury in wild-type mice (23). The authors found that a single injury, in un-anesthetized animals, produced notable short-term abnormalities in behavior similar to a post-concussive state. Repetitive injury (42 impacts total: 6 impacts per day over 7 days) produced chronic deficits, particularly with regard to depressive-like and risk-taking behaviors as well as spatial learning and memory (23). This same group published a recent follow-up study demonstrating the presence of hyperphosphorylated tau, a precursor of NFT development, in repetitively injured animals (20). The model produces several of the same behavioral symptoms reported by patients suspected of having CTE. We have recently shown that endoplasmic reticulum stress contributes to tauopathy and CTE-like behavioral deficits following repeat blast injury (24).

Other studies, conducted by Mouzon and colleagues report both behavioral and neuropathological changes following repetitive neurotrauma in mice. Specifically, five injuries administered 48 h apart, produced durable cognitive deficits, learning disabilities, diminished rotarod performance, and changes in anxiety-like behavior on EPM. Notably, these behavioral changes occurred in conjunction with persistent neuroinflammatory changes and disruption of white matter integrity. No changes in Aβ and tau phosphorylation were seen at the chronic time points of 6 and 12 months post-neurotrauma, likely because rodents do not naturally develop NFTs (25). Rodents do, however, demonstrate acute tau changes due to phosphorylation and cleavage following injury (26). Liu and colleagues found that tau hyperphosphorylation was increased in rats acutely post-injury and triggers caspase activation in rat cortices (27). The activation of cell death can lead to circuit dysfunction and behavioral deficits (28). Goldstein and colleagues show that tauopathy contributes to mitochondrial dysfunction and microtubule injury that ultimately leads to apoptosis. In this study, they found that tau modulation is a potential avenue for therapeutic intervention (29). We have recently reported that caspase activation is increased in human CTE brains near sites of NFT formation (30).

Other repeat-injury studies have investigated changes in tau and amyloid post-injury with the goal being to more clearly elucidate the relationship between TBI- and neurotrauma-related neurodegeneration. One report showed an increase in neuronal tau immunoreactivity (31) and another showed elevated amyloid precursor protein (APP) (13) at a variety of time points post-injury. A final study by Zhang and colleagues showed that monoacylglycerol lipase can lead to behavioral deficits and tauopathy characteristic of a CTE-like phenotype (32). These findings were further verified in other studies using transgenic models of amyloidosis and tauopathy in which repetitive injury paradigms produced elevated amyloid and tau levels with increased deposition. Single injury, however, failed to produce changes above control levels (33, 34). Using a T44 tau Tg mouse line, Yoshiyama and colleagues sought to study *dementia pugilistica* (DP), a condition sharing many features with modern day CTE, but with several key differences. DP by definition occurs only in boxers and has more severe gross anatomical changes that are not always present in CTE. The Yoshiyama group found that four impacts per day given once a week for 4 weeks produced only modest neuropathology with only one mouse demonstrating CTE or DP characteristics out of a total of 18 Tg mice and 24 wild-type mice (13, 35). The affected mouse displayed neuropathological changes that included heightened tau burden, the presence of NFTs, and cognitive deficits (35). While it remains unclear why only one mouse developed such pathology, neuropathological findings from this one animal demonstrated that iron deposition was increased and associated with blood–brain barrier disruption. Iron deposition, associated with degradation of heme, activates oxidative stress-related pathways. Importantly, this oxidative stress is then associated with accelerated NFT formation in these perivascular locales (35).

Notably, even single-injury paradigms have been shown to produce tauopathy in a variety of injury models, including blast (17), fluid percussion (36), and controlled cortical impact, findings consistent with prior clinical reports (37, 38). Goldstein and colleagues demonstrated that tauopathy following single blast injury was associated with hippocampal-dependent learning and memory deficits at subacute and chronic time points. These changes were also associated with electrophysiological alterations in long-term potentiation (LTP) (17). Therefore, even a single mild to moderate injury may induce neurodegeneration and neurological deficits leading to impaired cognition and disrupted synaptic transmission (17). Single injury has also been shown to contribute to blood–brain barrier disruption (39). This is a significant finding considering the numerous concussive and subconcussive injuries occurring in athletics and on the battlefield. Other studies using single-injury models have also demonstrated activation of numerous pathological processes and behavioral changes associated with neurodegeneration. Modeling neurotrauma-related neurodegeneration is a key component in the search for a model of CTE (40–42). While the importance of repeat injury in CTE modeling cannot be overstated, some single-injury studies have led to advances in the ability to detect phospho-tau in serum at weeks to months post-injury. These advances indicate the potential role of biomarkers in monitoring and understanding disease pathophysiology (38). The development of animal-based models that exhibit similar characteristics of CTE will afford researchers the opportunity to characterize the acute and chronic effects of injury on the phosphorylation of tau in controlled experimental conditions. *In vitro* models may even be beneficial in elucidating changes at the cellular level (43). Evaluating imaging modalities, potential biomarkers, such as phosphorylated tau, and proposed therapeutics in a controlled context will promote advancement toward clinical applications and could be instrumental for monitoring and understanding disease pathophysiology in the future.

## Implications for Modeling CTE: Learning from Past Shortcomings with TBI Models

Despite these notable advancements in CTE modeling as outlined above, the vast majority of repeat-injury studies fail to address the role of injury severity, inter-injury interval, and the total number of impacts needed to reproduce a CTE-like state (44, 45). Most studies simply describe features of TBI without relating the findings to neurodegeneration (46). Furthermore, because CTE is a neuropathological diagnosis, any study claiming to serve as a model of CTE must demonstrate the hallmark neuropathological changes (tau hyperphosphorylation and NFTs) and show that these changes persist at delayed time points and coexist with behavioral deficits. Few studies have looked at amyloid, tau, or TDP-43 accumulation following injury.

One of the most common omissions from repetitive injury paradigms is the lack of consideration for inter-injury interval. Work by Longhi and colleagues directly explored this issue, demonstrating that in a mouse model of closed-head injury (CHI), mice had a period of vulnerability estimated between 3 and 5 days where the effect of injuries was additive and produced deficits in cognition. When the inter-injury interval was lengthened to 7 days, these deficits were not present (47). While the focus of that study was elucidating differences in the inter-injury interval, other groups employing a repeat-injury paradigm should consider how the inter-injury interval may relate to their findings. Inter-interval design will be an important consideration in developing CTE models. Attempting to provide a clinical context for these decisions would be desirable, with the interval between severe concussive-type impacts ideally being longer than that between mild subconcussive-type impacts. This would expand scientific insight for “return-to-play” guidelines that require players to be asymptomatic following a diagnosed concussion prior to returning to contact sports. These types of questions have generally not been considered in preclinical work, with most studies using brief inter-injury intervals, often ranging from minutes to 24 h with poor justification for timing (23, 48–52).

Another important limitation, related to interpretation of injury severity and neuropathological outcome, is the sensitivity of detecting neuropathology using current scientific approaches. Shitaka and colleagues demonstrated previously that silver staining was more reliable for detecting axonal injury and pathology in comparison to routine histological analysis, assessment of neuronal cell loss, and APP immunohistochemistry (49). It may be increasingly important, particularly in studies of repetitive subconcussive impacts, to utilize measures of high sensitivity for injury detection. Silver staining, which has been shown repeatedly to exhibit a higher degree of sensitivity in detecting axonal injury than many immunohistochemical techniques, electron microscopy, or other markers with these characteristics may prove promising (49). It has not yet been determined if NFTs accumulate around sites of axonal shearing.

## Key Questions Going Forward

Improving experimental models will enhance the quest for developing therapeutic agents that can be used to prevent and treat CTE. The search for a model of CTE raises a number of questions that are important clinically. These questions include issues such as length of the inter-injury interval, the number and severity of impacts, and the age at time of impacts, as well as the mechanism of impact, the gender of the patient, and what role genetic predisposition may play in the development of neurodegenerative disease following neurotrauma. Another important question that must be addressed is does a history of neurotrauma and potential presence of CTE accelerate the development of AD? Furthermore, how can the period of susceptibility following neurotrauma be identified most readily? In the following sections, we attempt to address these questions based on available evidence. We also provide suggestions for handling shortcomings going forward. Studies on which these sections are based are referenced in Tables 1–7 for quick reference.

**Table 1:** **Chronic traumatic encephalopathy TBI models**.

Study	Sex/species/age	Model	Injuries	Interval	Anesthesia	Outcome measures
Liu et al. (27)	Male S. Dawley rat 2–3 months	Metal CCI Open head	Single	Single	Isoflurane	Tauopathy; cell death; apoptosis
Goldstein et al. (17)	Male C57BL6 mice 2–3 months	Blast Closed head	Single	Single	Ketamine/xylazine	Electrophysiology; tauopathy; axonal damage; motor; cognition; structural integrity; advanced imaging; human studies
Ojo et al. (18)	Male/female C57BL6 mice, hTau Tg mice 18 months	Metal CCI Closed head	Single Repeat (5)	Single (48 h)	Isoflurane	Tauopathy; gliosis and degeneration; structural integrity; cell death
Mouzon et al. (25)	Male C57BL6 mice 9–15 months	Metal CCI Closed head	Single Repeat (5)	Single (48 h)	Isoflurane	Motor; cognition; anxiety; inflammation; tauopathy; axonal damage
Huber et al. (26)	Male C57BL6 mice 2–3 months	Blast Closed head	Single	Single	Isoflurane	Motor; oxidative stress; tauopathy
Luo et al. (22)	Male C57BL6 mice, GFAP_Luc_ mice 2–3 months	Rubber CCI Closed head	Single Repeat (2, 3, 5)	Single (24 h)	Isoflurane	Bioluminescence; motor; anxiety; cognition; fear conditioning; gliosis and degeneration; apoptosis
Glushakova et al. (39)	Male S. Dawley rats 2–3 months	Metal CCI Open head	Single	Single	Isoflurane	Vascular and axonal damage; gliosis and degeneration
Zhang et al. (32)	Male C57BL6 mice 2–3 months	Metal CCI Closed head	Single Repeat (3)	Single (24 h)	Avertin	Electrophysiology; neuroscore; inflammation; tauopathy; gliosis and degeneration; cognition
Kondo et al. (29)	Male C57BL6 mice 2–3 months	Single blast and weightdrop Closed head	Single Repeat (7)	Single severe Seven mild over 9 days	Isoflurane	Electrophysiology; motor; cognition; anxiety; structural integrity; axonal damage; tauopathy; cell death; mitochondrial function; human studies
Lucke-Wold et al. (24, 30)	Male S. Dawley rats 2–3 months	Blast Closed head	Single Repeat (6)	Single Six mild over 10 days	Isoflurane	Cognition; endoplasmic reticulum stress; tauopathy; human studies

**Table 2:** **Shock tube TBI models**.

Study	Sex/species/age	Model	Injuries	Interval	Anesthesia	Outcome measures
Long et al. (74)	Male S. Dawley rats 2–3 months	5.3-m metal tube	Single	Single	Isoflurane	Cardiovascular; motor; cognition; structural integrity; vascular damage; degeneration
Budde et al. (75)	Unknown S. Dawley rats Unknown	3.3-m metal tube	Single mild and severe	Single	Isoflurane	Advanced imaging; anxiety; cognition; gliosis and degeneration; apoptosis
Genovese et al. (50)	Male S. Dawley rats 2–3 months	5.3-m metal tube	Repeat (3)	24 h	Isoflurane	Fear conditioning
Wang et al. (52)	Male C57BL6 mice 2–3 months	5.3-m metal tube	Repeat (3)	1 or 30 min	Isoflurane	Mitochondrial function; DNA fragmentation; righting reflex; apoptosis
Lucke-Wold et al. (6, 42)	Male S. Dawley rats 2–3 months	0.3-m metal tube	Single	Single	Isoflurane	Vascular damage; structural integrity; gliosis and degeneration
Logsdon et al. (41)	Male S. Dawley rats 2–3 months	0.3-m metal tube	Single	Single	Isoflurane	Vascular damage; endoplasmic reticulum stress; cell death; apoptosis; anxiety

**Table 3:** **Weight-drop TBI models**.

Study	Sex/species/age	Model	Injuries	Interval	Anesthesia	Outcome measures
DeFord et al. (72)	Male C57BL6 mice 2–3 months	Weight-drop Closed head	Single Repeat (4)	Single (24 h)	Isoflurane N_2_O and O_2_ (70:30)	Neuroscore; cell death; vascular damage; cardiovascular; cognition
Creeley et al. (48)	Male C57BL6 mice 2–3 months	Weight-drop Closed head	Repeat (3)	24 h	Isoflurane	Motor; cognition; righting reflex; cell death
Fujita et al. (53)	Male S. Dawley rats 3–6 months	Weight-drop Skull-exposed	Single Repeat mild (2, 3) Repeat medium (2) Repeat severe (2)	Single Two mild over 3 h Three mild over 2 h Two medium over 3 h Two severe over 3 h Two severe over 5 h Two severe over 10 h	Pentobarbital	Vascular reactivity to ACh; axonal damage
Meehan et al. (54)	Male C57BL6 mice 2–3 months	Weight-drop Closed head	Single Repeat daily (3,10) Repeat variable (5)	Single Daily (3, 5, or 10) Weekly (5) Monthly (5)	Isoflurane N_2_O and O_2_ (70:30)	Edema; axonal and vascular damage; cell death; cognition
Mannix et al. (54)	Male C57BL6 mice 2–3 months	Weight-drop Closed head	Single Repeat daily (5,7) Repeat variable (5)	Single Daily (5 or 7) Weekly (5) Biweekly (5) Monthly (5)	Isoflurane N_2_O and O_2_ (70:30)	Cognition; tauopathy; advanced imaging
Weil et al. (57)	Male Swiss web. mice 2–3 months	Weight-drop Skull-exposed	Single Repeat (2)	Single 3 or 20 days	Isoflurane	Glucose metabolism; inflammation; gliosis and degeneration; cell death; cognition

**Table 4:** **TBI models not using craniotomy**.

Study	Sex/species/age	Model	Injuries	Interval	Anesthesia	Outcome measures
Mouzon et al. (65)	Male C57BL6 mice 2–3 months	Metal CCI Closed head	Single Repeat (5)	Single (48 h)	Isoflurane	Motor; cognition; gliosis and degeneration; righting reflex; axonal damage
Yoshiyama et al. (35)	Male/Female B6D2/F1 mice, Tau Tg mice 12 months	Silicone CCI Skull-exposed	Repeat (16)	Four per day Every 20 min Once a week for 4 weeks	Isoflurane	Neuroscore; cognition; gliosis and degeneration; tauopathy
Laurer et al. (64)	Male C57BL6 mice 2–3 months	Rubber CCI Skull-exposed	Single Repeat (2)	Single (24 h)	Pentobarbital	Neuroscore; motor; cardiovascular; cognition; axonal and vascular damage; cell death; tauopathy
Bolton and Saatman (56)	Male C57BL6 mice 2–3 months	Silicone CCI Skull-exposed	Single Repeat (5)	Single (24 or 48 h)	Isoflurane	Cardiovascular; righting reflex; axonal damage; gliosis and degeneration; tauopathy
Shitaka et al. (49)	Male C57BL6 mice 2–3 months	Rubber CCI Skull-exposed	Repeat (2)	24 h	Isoflurane	Cognition; structural integrity; gliosis and degeneration; axonal damage; electron microscopy
Klemenhagen et al. (51)	Male C57BL6 mice 2–3 months	Rubber CCI Skull-exposed	Repeat (2)	24 h	Isoflurane	Fear conditioning; cognition; social recognition; depression; anhedonia; gliosis; vascular damage
Uryu et al. (33)	Male/Female B6D2/F1 mice, APP Tg mice 9–12 months	Rubber CCI Skull-exposed	Single Repeat (2)	Single (24 h)	Pentobarbital	Neuroscore; cognition; motor; vascular damage; gliosis and degeneration; tauopathy; oxidative stress
Longhi et al. (47)	Male C57BL6 mice 2–3 months	Silicone CCI Skull-exposed	Single Repeat (2)	Single (3, 5, or 7 days)	Isoflurane	Cognition; motor; righting reflex; gliosis and degeneration; axonal and cytoskeletal damage; cell death; edema
Conte et al. (34)	Female B6D2/F1 mice, APP Tg mice 9–12 months	Rubber CCI Skull-exposed	Repeat (2)	24 h	Isoflurane	Cognition; tauopathy; structural integrity; oxidative stress

**Table 5:** **TBI models using craniotomy**.

Study	Sex/species/age	Model	Injuries	Interval	Anesthesia	Outcome measures
Olsson et al. (68)	Rabbits Unknown	Fluid percussion	Single Repeat (10)	Single (5 min)	Pentobarbital	Righting reflex; cardiovascular; vascular damage
Smith et al. (40)	Male C57BL6 mice 2–3 months	Metal CCI Open head	Single	Single	Pentobarbital	Cognition; structural integrity; cell death; gliosis and degeneration; vascular damage
Kanayama et al. (31)	Male Wister rats 2–3 months	Fluid percussion	Single Repeat (7)	Single (24 h)	Pentobarbital	Motor; social recognition; cytoskeletal damage; tauopathy
Allen et al. (158)	Male S. Dawley rats 2–3 months	Weight-drop Plexiglas piston	Single severe Repeat mild (3)	Three mild over 14 days ± severe 3 days	Pentobarbital or ketamine/rhompamine	Motor; gliosis and degeneration; structural integrity
DeRoss et al. (70)	Male Long–Evans rats 2–3 months	Fluid percussion	Single Repeat (2,3)	Single (N/A)	Isoflurane	Cognition; motor
Manley et al. (73)	Male Yorkshire pigs Adult	Metal CCI Open head	Single	Single	Pancuronium Isoflurane	Cardiovascular; structural integrity; edema; vascular damage; cell death; gliosis and degeneration
Donovan et al. (61)	Male S. Dawley rats 2–3 months	Metal CCI Open head	Single Repeat (2)	Single 7 days each side	Isoflurane	Advanced imaging; structural integrity; axonal damage
Hawkins et al. (61)	Male S. Dawley Rats 6–8 months	Fluid percussion	Single	Single	Isoflurane	Extensive tauopathy assessment
Rubenstein et al. (38)	Male S. Dawley rats and C57BL6 mice 2–3 months	Metal CCI Open head	Single	Single	Isoflurane	Extensive tauopathy assessment; human studies
Begum et al. (37)	Male S. Dawley rats 2–3 months	Metal CCI Open head	Single	Single	Isoflurane	Motor; endoplasmic reticulum stress; tauopathy; axonal damage
Aungst et al. (67)	Male S. Dawley rats 2–3 months	Fluid percussion	Single Repeat (3)	Single (48 h)	Isoflurane	Electrophysiology; neuroscore; cognition; social recognition; gliosis and degeneration; cell death

**Table 6:** **Other *in vivo* TBI models**.

Study	Sex/species/age	Model	Injuries	Interval	Anesthesia	Outcome measures
Raghupathi et al. (69)	Male Farm pigs 3–5 days	Non-impact head rotation	Single Repeat	Single (15 min)	Isoflurane	Cardiovascular; axonal and vascular damage; structural integrity; cell death
Friess et al. (58)	Male Farm pigs 3–5 days	Non-impact head rotation	Single Repeat (2)	Single (24 h or 7 days	Isoflurane	Motor; cognition; axonal damage
Roth et al. (77)	Male S. Dawley rats and C57BL6 mice 2–3 months	Skull thinning compression Skull-exposed	Single	Single	Ketamine/xylazine/acepromazine	Inflammation; gliosis and degeneration; oxidative stress; vascular damage; structural integrity; advanced imaging; human studies
Petraglia et al. (20, 23)	Male C57BL6 mice 2–3 months	Rubber CCI Helmet	Single Repeat (42)	Six per day every 2 h over 7 days	No Anesthesia	Neuroscore; motor; cognition; anxiety; depression; sleep

**Table 7:** ***In vitro* and *ex vivo* TBI models**.

Study	Model	Cell Line	Injuries	Interval	Severity	Outcome measures
Zander et al. (43)	Primary blast	PC12 neurons	Single Repeat (3)	Single Three over 20 min	Mild to moderate	Membrane permeability; cell viability; axonal damage
LaPlaca and Thibault (78)	Shear stress	Neuronal culture	Single	Single	Mild	Membrane permeability; calcium influx; cell death
Morrison et al. (81)	Membrane strain	Organo-typic hippocampal slices	Single	Single	Mild to Severe	Cell death; apoptosis; membrane permeability
Mukhin et al. (79)	Blade transection	Neuron/glial culture	Single	Single	Severe	Cell death; excitotoxicity
Sieg et al. (80)	Mechanical compression	Organo-typic cortical slices	Single	Single	Severe	Cell death; apoptosis; axonal damage
Slemmer et al. (63)	Cell stretch	Neuronal culture	Single Repeat (6)	Single Six over 24 h	Mild	Cell viability; cell death

### Inter-Injury Interval

The effect of inter-injury interval on outcome following TBI has only recently been investigated. Studies have explored a variety of intervals ranging from 2 min apart (*in vitro*) to a few hours (*in vivo*) to as long as 30 days apart (*in vivo*). Using a novel approach assessing vasoreactivity in TBI, Fujita and colleagues demonstrated that administration of seemingly mild injuries at brief intervals (3 h apart) produced dramatic declines in vasoreactivity and axonal pathology. When the inter-injury interval was lengthened to 5 h, the magnitude of these changes was diminished substantially with complete dissolution of changes in both pathology and vasoreactivity at 10 h (53).

Other studies that have investigated longer inter-injury intervals have identified periods of susceptibility following an initial impact at periods ranging from 24 h to a few weeks (54–57). Bolton and colleagues demonstrated with a CHI model that a single impact produced extensive gliosis bilaterally in the hippocampi and entorhinal cortices. Repeat injury after 24 h produced a more severe injury consisting of hemorrhage in the entorhinal cortices as well as heightened measures of neurodegeneration, gliosis, and neuroinflammation (56). When the experimental paradigm was changed such that impacts were given with a 48 h inter-injury interval, the histopathology resembled that of a single impact suggesting enhanced susceptibility when a second impact was administered within 24 h (56).

Mannix and colleagues performed one of the most rigorous investigations of the effect of inter-injury interval on outcomes associated with TBI. These studies included measures of both cognition and neuropathology related to the development of neurodegenerative diseases such as CTE and AD. In this study, the investigators showed that animals that received daily or weekly injuries with weight-drop had persistent cognitive deficits up to 1 year post-injury (55). This was in contrast to when animals were injured biweekly or monthly, which failed to produce deficits at such a chronic time point (55). Notably, the cognitive deficits seen in the daily- and weekly injured animals did not correlate with elevations in tau phosphorylation or amyloid-β when measured by ELISA nor brain volume loss when measured by MRI (55). This finding may indicate that in addition to inter-interval time, injury severity must be considered. Tauopathy is essential for modeling CTE therefore an appropriate inter-injury interval might be best characterized in transgenic animals. Weight drop produces variable injury based on the height of the drop. A more severe TBI can produce cognitive deficits but may not be representative of the concussive and subconcussive injuries associated with CTE. A mild TBI with a transgenic animal will likely produce the most relevant deficits.

Meehan and colleagues performed a similar study with primarily behavioral assays. The investigators subjected mice to a CHI via weight-drop for a total of five impacts at various intervals. These intervals included daily, weekly, and monthly intervals. Mice receiving five impacts total at daily or weekly intervals were impaired in the MWM compared to sham animals (54). This was not the case when injuries were delivered at monthly intervals, as these animals exhibited no impairment in the MWM (54). Interestingly, at 1 month post-injury, the daily- or weekly injured animals still exhibited deficits in the MWM and this deficit persisted in daily-injured animals out to 1 year (54). This finding may represent why football lineman who experience daily subconcussive injuries appear more likely to develop CTE based on the clinical cases reported. It is still necessary to establish if tauopathy is the driving mechanism behind behavior. The findings confirm and expand upon the inter-interval studies completed by Longhi and colleagues (47). Longhi reported that shorter inter-interval injuries produce worse outcomes, which is in agreement with the Mannix and Meehan findings. These studies were consistent with findings in higher phylogenetic species as well, specifically piglets. Friess and colleagues showed that a 24-h inter-injury interval produced more severe deficits and higher mortality rates than when the interval was extended to 7 days (58). Finally, Kanayama and colleagues demonstrated a graded response in locomotor activity. Both shorter inter-injury intervals and greater number of total injuries were associated with worse outcome (31).

Weil and colleagues explored the effect of altering the inter-injury interval in relation to recovery from TBI. They used a clear clinical-minded approach and utilization of metabolic imaging (PET). This group showed that injuries separated by only 3 days were associated with worse neuropathology and an inability to mount the typical hypermetabolic response with regard to glucose utilization following TBI. This worse outcome was not seen following either a single injury or repeat injuries with an extended inter-injury interval of 20 days (57). Similarly, a brief inter-injury interval of 3 days was associated with elevated IL-1β and TNFα gene expression when compared with other experimental groups (57).

The longest interval between injuries used in preclinical studies, to the best knowledge of the authors, was 30 days. The additional injury had no additive effect on anxiety (EPM), depression (FST), and cognitive function (MWM) when compared to animals receiving only one injury (59). These findings indicate that either the window of vulnerability following the first injury was avoided or that the response to the first injury may protect the animal from subsequent injuries, a concept known as preconditioning (59). The progression toward tauopathy was not well characterized in this work.

*In vitro* studies have shown similar findings to the *in vivo* studies described above. Shorter inter-injury intervals between mechanical stretching resulted in an elevation in S-100β protein release and increased cellular permeability identified with propidium iodide staining (13). Similarly, a “subthreshold” level of stretch did not produce any overt cellular damage or death when repeated at 1 h intervals but did cause neuronal loss and neuron-specific enolase (NSE) release when performed at incredibly short intervals (every 2 min) (13, 60). Remarkably, this rapid and repetitive “subthreshold” stretch that produced changes in neurons, failed to produce an increase in S-100β protein release, indicating a differential response between neurons and glia to neurotrauma severity and interval (13, 60).

In contrast to the above studies, one group showed that repeat injury, when administered in different anatomical locations within the brain, failed to result in heightened damage when an inter-injury interval was 3 days. It did, however, increase tissue vulnerability with a 7-day interval as evident by increased hemorrhage (61, 62). The authors of the study therefore argue that the period of susceptibility likely depends on not only the time interval between injuries but also the anatomic location of injury (61, 62). Importantly, this study utilized an open injury model (controlled cortical impact), a scenario that is only seen in a subset of clinical neurotraumas.

In summary, these studies demonstrate a period of vulnerability following initial injury in which sustaining a second brain injury may result in an additive effect. Additive injury is not clearly apparent when the brain is allowed a more extensive recovery period. Interestingly, this vulnerability may not solely be due to initial axonal pathology but may also be the result of cerebrovascular reactivity and the inability to utilize glucose effectively (53, 57). This concept of a varied cellular response to TBI is consistent with findings from *in vitro* studies that demonstrate a varied response amongst glia and neurons (13, 60, 63).

### Number of Impacts

The prevailing theory for “mild” neurotrauma is that repetitive injuries are associated with more short- and long-term detrimental effects than a single injury alone (56, 64, 65). This concept has been applied regardless of injury severity (concussive versus subconcussive) with emerging evidence indicating that even subthreshold impacts are cumulatively detrimental (53, 66). In this section, we explore the effect of repeat injury through analysis of studies employing both repeat and single-injury paradigms.

One of these studies, conducted by Mouzon and colleagues, showed that when rodents are exposed to five total injuries with an interval of 48 h, these animals exhibit both impaired learning and memory at extended time points (25). These findings are in contrast to single-injured animals that display only learning deficits but no retention impairment at the same time points (25). This study closely parallels the clinical findings documented by Guskiewicz and colleagues in which former athletes with a history of repetitive concussions experience memory-related issues at a rate of five times higher than those without a history of concussion (12, 25).

Other work that investigated various iterations of impacts (0, 1, 3, 5, and 10) demonstrated that while a single injury does not produce deficits in MWM performance in comparison to sham-injured animals, repetitive injury does in fact produce deficits and these deficits may exhibit a dose-dependent relationship. When mice were given 10 concussive weight-drop injuries, those in which the weight was dropped from a height of 42″ performed worse than those injured from a height of 38″. Therefore, this work demonstrates the potential for both injury number and injury severity in contributing to neurological dysfunction (54). Luo and colleagues utilized a GFAP-driven luciferase mouse line and a repetitive closed-head injury model to investigate cumulative decline. The investigators showed that there appeared to be a linear increase in GFAP luminescence from 1 to 3 injuries but that this response appeared to reach a plateau by five injuries (22). In addition to the increase in GFAP fluorescence with repetitive injuries, mice receiving three injuries demonstrated less freezing time than sham animals in both cued and contextual fear conditioning (22). This was in contrast to single-injury animals that did not differ from sham-injured animals in cued or contextual memory (22). Consequently, these data demonstrate that an increase in injury number is associated with an increasing severity of injury markers (based on protein expression of GFAP) as well as functional deficits (fear conditioning). Others employing both single- and repeat-injury paradigms have shown that while single injury may not induce pathological findings, repeat injury, of the same severity, does. This was particularly notable in work by Uryu and colleagues in which repetitive injury produced an increase in Aβ deposition in Tg2576 animals, whereas no increase was observed in single-injury paradigms in these same animals (33). Likewise, Kanayama and colleagues demonstrated that repeat-injury paradigms induced tau hyperphosphorylation, a precursor to NFT formation, in conditions such as CTE and AD (31).

The observed findings in closed-head, repeat-injury models, were also consistent with those found in open-head injury using a craniectomy and controlled cortical impact. In work by Donovan and colleagues, the investigators showed that repeat injury induces progressive and evolving changes that are not observed in single-injury paradigms (62). The basis of many of the memory and cognitive changes following repeat TBI may be explained by electrophysiological alterations in synaptic transmission. In work by Aungst and colleagues, repetitive TBI was found to prevent the induction of LTP 28-days post-injury due to alterations in the NMDA receptor. This is in stark contrast to single-injury paradigms that revealed the ability to induce LTP in both hemispheres, with the contralateral hemisphere exhibiting less LTP than the ipsilateral hemisphere. The impairments in excitatory neurotransmission following repeat injury were accompanied by extensive neuroinflammation and neurodegeneration as well as behavioral/functional impairments (67). Specifically, repeat injury produced deficits more severe than single injury when measured at 1-week intervals out to a month. Importantly, even single injury produced deficits at chronic time points post-injury when compared to sham animals, indicating long-term effects of TBI (67).

Povlishock and colleagues expanded the concept of subthreshold injury. In these studies, the investigators showed that administering a single weight-drop injury from 1.0 m resulted in neither axonal nor microvascular change. With repeat injury of short inter-injury intervals (hours), significant axonal and microvascular pathology was observed (53). This work was the first to assess microvascular reactivity to acetylcholine (ACh) following repetitive subthreshold brain injury. This work demonstrates the clear danger of subthreshold impacts when sustained in a repeated and rapid fashion. It also illustrates the role of the microvasculature in neuronal injury, showing that the neurovascular unit is essential for neuronal homeostasis.

Importantly, work regarding the number of impacts has been extended higher up the phylogenetic tree to rabbits and piglets (68, 69). In rabbits, repeated loading with loads of up to 1.5 atm failed to produce an additive concussive response over a single load. A multi-loading paradigm at higher loads, however, caused respiratory arrest (68). In piglets, multiple less severe injuries induced neuropathological findings similar to a severe load based on the density of injured axons as well as number and distribution of foci (69).

*In vitro* studies have reported similar findings to the *in vivo* studies. Weber and colleagues applied a mild stretch to hippocampal neuronal cultures that produced low-grade injury when applied at a single time. When this injury was repeated, the cells exhibited cumulative damage with two injuries inducing an increase in NSE (13, 60, 63).

Notably, the group led by Mychasiuk and colleagues provides evidence contrary to the widely held belief that an increased number of impacts are associated with detrimental findings on behavioral or histological measures. With a 30-day inter-injury interval, rodents receiving multiple injuries performed similar to single-injury animals on measures of anxiety, depression, and cognitive ability. Therefore, they propose that receiving head injury at an early age may prime the brain to be less susceptible to the effects of a later neurotrauma, a theory known as “preconditioning” (59). DeRoss et al. also showed that while one concussive impact resulted in diminished performance in 85% of animals, less deviation was seen with subsequent impacts. The number of impacts has an inverse relationship with animal performance in the water maze (70). Again, the mechanism behind these findings is not entirely clear as those animals sustaining multiple injuries also received additional exposure to the water maze, allowing for enhanced training/learning of the maze (70). Therefore, while repetitively injured animals did better than single-injured animals in the maze, this is likely a product of increased training rather than a protective response but this cannot be said with absolute certainty.

### Severity of Impacts

The effect of injury severity on likelihood of neurodegenerative disease development is not entirely clear, although some clinical reports indicate that more severe injury results in a greater predisposition for AD development (33, 71). What is known from preclinical studies using an array of animal models is that there is a dose-dependent increase in neural injury markers and cognitive deficits with more severe injury (22, 72–76). Similarly, repetitive “mild” injuries may produce a phenotype more consistent with a single more severe injury (72).

How “mild” TBI contributes to the likelihood of developing CTE remains unclear. Emerging evidence from preclinical studies raises concern about lasting effects of subthreshold injuries when sustained in a rapid and repetitive fashion. These repetitive injuries contribute to vascular reactivity and subsequent axonal degeneration *in vivo* (53). Some studies suggest that severity of injury may dictate the rest period required to minimize cumulative cognitive deficits, although further studies are required to validate these findings (54).

Similarly, preclinical studies, even those in which only a mild force is imparted to a thinned cranium, indicate that a substantive inflammatory response is produced quickly after injury (77). This response is associated with heightened vascular permeability, also seen clinically, as well as microglial response (77). It is these mechanisms, both primary and secondary, that may contribute to neurodegenerative disease post-neurotrauma.

*In vitro* studies may be of further use in addressing the role of injury severity, particularly in subconcussive/subthreshold-type studies, as levels of injury and subsequent cellular responses can be monitored rapidly and performed over a greater number of iterations at a lower cost in comparison to *in vivo* studies. In fact, a number of injury paradigms and mechanisms have been investigated successfully in this manner, including fluid pulse-induced shear stress, repetitive stretching, and other mechanical deformation procedures, both *in vitro* and *ex vivo* (60, 63, 78–81). The *in vitro* shearing studies found a significant amount of axonal beading and glial death (82).

### Age at Time of Impacts

Age is the biggest risk factor for the development of neurodegenerative disease and has been associated with poor outcomes following TBI in a variety of clinical and preclinical reports (83–91). Similarly, neurotrauma has been associated with an increased risk of neurodegenerative disease development with regard to AD (71, 92–94), PD (95), and CTE (2, 5, 7, 16, 17, 96–103). One of the primary questions currently in the field is how the age at which the patient sustains the neurotrauma pertains to the development, or lack thereof, of CTE.

Similar findings have been observed in preclinical studies. Mychasiuk and colleagues showed that TBI during brain development leads to worse outcomes than TBI affecting the mature brain (59). Preclinical work has also shown that young animals experience less edema than middle-aged animals following TBI (104). The increased edema is associated with an increase in lesion size in aged rodents experiencing TBI (105). TBI in aged rodents is also more likely to increase sensorimotor and cognitive decline (85). TBI in youth may ultimately be more detrimental for social development, whereas severe injury in the elderly results in rapid cognitive decline due to increased edema and therefore, lesion size. In regard to human TBI, it is unclear if the elderly would have a more progressive form of the disease similar to rodent studies or if the disease would develop in the normal manner. It is also important to consider that males <35 years old are the most likely to have repetitive TBIs, a group associated with heavy participation in sports and now the military (106). Future preclinical studies should therefore continue to investigate TBI secondary mechanisms in both young and aged animals with particular attention paid to addressing repetitive injury paradigms and the development of CTE-like features, both behaviorally and biochemically.

### Mechanism of Impact

Chronic traumatic encephalopathy has been diagnosed in athletes sustaining direct impacts as a result of participation in warfare, football, wrestling, and soccer (2, 5, 7, 16, 17, 96–103). The mechanism of injury is different between blast and athletic concussions, but how these mechanisms relate to injury progression remains to be elucidated. For blast, primary to quaternary injury must be considered (107). Other important questions include what is the influence of linear versus rotational impacts? What is the effect of direct impacts such as a football tackle versus indirect impacts such as primary blast exposure? Furthermore, how can comparisons most accurately be made across these various impacts? Are accelerometers and recording systems (such as the HITS system) the best method for understanding and comparing these impacts? What role does high-speed videography and subsequent kinematic analysis play? Each of these questions remains to be answered and may provide further insight into understanding the role that impact type plays in CTE development.

### Biochemical Mechanisms

Interestingly, glymphatic clearance has recently been shown to play a role in injury progression. Iliff and colleagues showed increased tauopathy accumulation in aquaporin knock-out mice following traumatic brain injury due to disrupted glymphatic clearance (108). It has yet to be determined how the primary injury mechanism causes the disruption to glymphatic channels. Cernak proposed an interesting theory about low-frequency stress waves transmitting kinetic energy through tissue (109). This mechanism may account for the dysfunction of the aquaporin channels. The energy transfer may also injure other cellular components such as axons or vessels. Chodobski and colleagues have shown that kinetic transfer of energy can account for blood–brain barrier disruption (110). Blood–brain barrier disruption post-injury can trigger increased neuroinflammation. Agoston and colleagues showed that blast traumatic brain injury, in particular, causes persistent neuroinflammation, which leads to behavioral deficits (111). The neuroinflammation can also contribute to post-traumatic epilepsy and tauopathy (112). Investigating how tauopathy spreads is a topic warranting further investigation (113). Furthermore, Kobeissy and colleagues highlight in their recent review that underlying neuronal damage can cause lasting neuropsychiatric deficits such as post-concussion syndrome and post-traumatic stress disorder (107).

### Role of Genetics

Genetics and lifestyle choices may play a role in likelihood of sustaining a TBI and also the outcome following a TBI. Little to no evidence currently exists regarding lifestyle choices associated with TBI and only recently has genetic contribution to CTE been addressed. Specifically, it is well known in the human literature that the APOϵ4 allele is associated with worse outcome following TBI (114). APOϵ4 and APOϵ3 have also been implicated in the development of both CTE and AD (13, 115). The mechanism by which APOϵ4 worsens outcome following TBI is poorly defined (55), but targeted replacement of the allele in mice has allowed focused research into cholesterol metabolism and may lead to insights in the field of traumatic brain injury and subsequent neurodegeneration (116). Notably, a study by Maroon and colleagues recently demonstrated that there was no significant difference between ApoE4 carriers in a population of patients afflicted with CTE when compared to the general population, suggesting that perhaps ApoE may not represent a significant risk factor for CTE development, even in individuals exposed to neurotrauma (117).

Other genetic influences have also been observed but are currently limited to preclinical evidence. Rare genetic alterations such as mutation of the *CACNA1A* calcium subunit gene have also been shown to lead to poor TBI outcomes in human patients (118). Recent preclinical studies have shed light on other potential genetic factors that may influence TBI outcomes. Decreases in micro RNA 23a and 27a increase apoptosis following TBI in rodents (119). Deficient caveolin expression can exacerbate neuroinflammation post-TBI (120). The knock-in mouse APP696swe has accelerated deposition of Aβ following TBI (35). Emerging evidence also suggests dysfunctional mitochondrial genes following TBI such as *Fas, Apaf1*, and *Chp*. Interestingly, these genes become more dysfunctional and mutated with time after mild TBI (121). TBI can also induce DNA fragmentation leading to an upregulation of p53, a critical regulator of cell cycle (122). On the other hand, upregulation of insulin growth factor expression prior to TBI is associated with neuroprotection (123). Similarly, genetic regulation of aquaporin 4 channels can reduce edema formation following TBI in rodent models (124). Surprisingly, disruption or the *PARP1* gene offers protection against TBI hypoxia (125). Studying genetic risk factors for TBI is an area of growing importance and requires further investigation. Understanding factors that lead to increased or decreased TBI severity may also allow the development of novel pharmaceutics for the prevention of neurodegenerative disease.

An emerging area is the role of epigenetic modulation following TBI. Epigenetic markers are now being used preliminarily to predict recovery following injury (126). VandeVord and colleagues show enhanced methylation of DNA in the rat hippocampus following blast traumatic brain injury (127). These epigenetic changes are mediated by HDACs and DNMTs (128). Interestingly, the methylation changes are cumulative with repetitive injury (129). HDAC has been shown to contribute to GSK3β activation, which is a known tau kinase. When HDAC is inhibited, white matter damage is reduced (130). Epigenetic regulation has been tied to the development of post-traumatic stress disorder clinically (131). Targeting epigenetic regulation may therefore be a viable target in preventing tauopathy and behavioral deficits following traumatic brain injury.

### Influence of Gender

The influence of gender on outcome after TBI remains controversial, particularly in light of the few cases of CTE diagnosed in women. Some studies claim that females have better outcomes following neural injury (132), while others report no change (133, 134), or worse outcome (135). Estrogen treatment has shown improved outcomes in rodent models of neural injury (136–139), including TBI (140, 141). Female rodents exhibit better outcomes after neural injury as evidenced by increased neurotrophin production (140), decreased neuroinflammation (142), and better performance on motor tasks (143). Clinical evidence shows that female patients exhibit lower oxidative damage after TBI (144), which could be the result of a higher estrogen circulation following injury. Gender differences should be considered when conducting clinical trials for TBI therapy.

Interestingly, Dixon and colleagues found that TBI in female humans reduces estradiol in the CSF (145). The reduction of estradiol may have unique long-term effects because it is not yet known if this is transient or permanent. The limited amount of data about women with TBI has restricted the comparisons of injury between genders (146). The majority of traumatic brain injuries in women are subdural hematomas from falls. Elderly women with subdural hematomas tend to fair worse than the general TBI population as a whole (147). Menopause may therefore dampen the neuroprotective physiological properties mediated by estrogen post-injury. TBI in elderly women is also linked with earlier onset AD (148). Hormonal changes may also be a contributing factor to CTE, but this has yet to be verified. It is clearly based on the limited preclinical and clinical data that further investigation into the gender differences surrounding TBI outcome is warranted.

### Effect of Environment

Another area of investigation required for elucidation of factors influencing the likelihood and/or severity of CTE development following neurotrauma includes the effect of environment. Areas of particular interest include social support, diet, use of supplements, and use of drugs or anabolic steroids.

Good social support has been shown to decrease the likelihood of developing a postconcussional disorder following acute head injury (149). Strong family support is linked to better outcomes (150). Another important feature of environment is diet. While the effect of diet is well understood with regard to general health, there are limited studies relating diet and mental health, particularly in the context of traumatic brain injury. A preclinical study conducted by Mychasiuk and colleagues showed that high-fat diet in conjunction with TBI resulted in cumulative deficits on assessments of motor function, short-term working memory, and produced depressive-like effects compared to animals with normal diet and TBI (59). Similarly, administration of dietary supplements such as Vitamin E and DHA has been shown to improve outcomes from TBI in numerous studies (34, 151–154). The suggestion has been made previously, based on evidence that some cases of diagnosed CTE occurred in former athletes with a history of anabolic steroid use, that anabolic steroid use may predispose these athletes to CTE development. While this question has not been completely resolved in terms of studying markers of CTE, preliminary studies found no difference in APP expression post-TBI regardless of when anabolic steroids were used (155).

### Preconditioning

**Figure 2:**
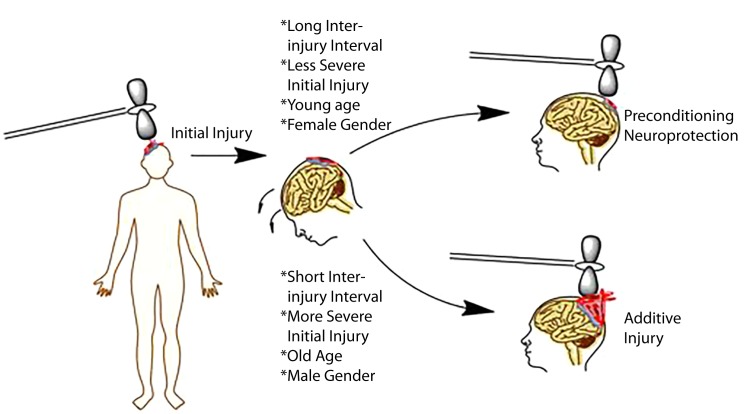
**Schematic representation of factors influencing injury outcomes, particularly in repetitive injury paradigms**. Initial evidence indicates that longer inter-injury intervals, less severe initial injury, a younger age, and female gender may serve as protective effects in repeat paradigms. By contrast, shorter intervals, increased severity, older age, and male gender may be associated with worse outcomes.

Any neural injury model has the potential to be complicated by the concept of preconditioning. Preconditioning at the most basic levels refers to neuroprotection for a given injury induced by a prior stimulus/injury. The concept of preconditioning has been well documented in a variety of neural injury models ranging from ischemic stroke to TBI (13). Initial stimuli that serve a protective effect in a subsequent injury include but are not limited to brief periods of ischemia, chronic exposure to moderate heat or heat acclimation, and subthreshold or mild injury (13). The proposed mechanism is that neuronal antioxidant machinery is upregulated with subthreshold injury thereby increasing the cells ability to respond to free radical production during subsequent injuries (156). Whether preconditioning serves a protective phenomenon in the development of CTE is unclear, but the general clinical consensus is that any brain injury no matter how small may be detrimental long term, minimizing any potential benefit of the preconditioning phenomenon (66, 157). This is in contrast to preclinical literature demonstrating that sustaining repetitive mild injury prior to a single severe injury protects the animal from the most deleterious effects of the severe injury (158). In other words, animals receiving repetitive mild injury prior to a severe injury do better than animals receiving a single severe injury (158). Similar findings have been observed *in vitro* in which subthreshold stretch prevented more deleterious injury when a threshold stimulus was given (60). The concept of preconditioning versus additive injury is captured pictorially in Figure 2.

## Summary

In conclusion, understanding CTE as a disease remains in its infancy and current studies remain largely speculative in nature without prospective clinical investigation. The required clinical studies to advance the field mandate extensive financial resources and time. Preclinical studies represent the most promising mechanism for studying many of the basic biologic questions about CTE, as discussed above. While these studies are continuing to evolve, numerous groups have reported exciting findings. Better modeling has allowed more extensive biochemical and behavioral characteristics to be defined. Now that our laboratory and others have established CTE models, options for translational investigation of CTE pathophysiology abound. In this work, we discussed numerous avenues for addressing translational questions, namely the role of (1) inter-injury interval, (2) number of impacts, (3) impact severity, (4) age at time of impacts, (5) mechanism of impact, (6) genetics, (7) gender, and (8) effect of environment on the development of CTE. We also highlighted some of the challenges of CTE modeling and specific requirements for successful models. By improving our understanding about CTE mechanisms, we believe that significant strides can be made not only in understanding CTE but also potentially developing prevention and therapeutic-related approaches. A companion manuscript describes our collective experience in modeling CTE, both neuropathologically and behaviorally.

## Conflict of Interest Statement

The authors declare that the research was conducted in the absence of any commercial or financial relationships that could be construed as a potential conflict of interest.
